# Novel imaging approaches for visualizing root–mycorrhizal fungal interactions

**DOI:** 10.1093/jxb/erag309

**Published:** 2026-07-03

**Authors:** Henry W G Birt, Stephen J Paisey, Patrick Möhl, Jasmine Hind, Jiaqi Xu, John Pickett, Matthew Tredwell, David Johnson

**Affiliations:** Department of Earth and Environmental Sciences, The University of Manchester, Michael Smith Building, Manchester M13 9PL, UK; Lancaster Environment Centre, Lancaster University, Lancaster LA1 4YQ, UK; Wales Research and Diagnostic PET Imaging Centre, Cardiff University, University Hospital of Wales, Heath Park, Cardiff CF14 4XN, UK; Department of Earth and Environmental Sciences, The University of Manchester, Michael Smith Building, Manchester M13 9PL, UK; Lancaster Environment Centre, Lancaster University, Lancaster LA1 4YQ, UK; School of Chemistry, Cardiff University Main Building, Park Place, Cardiff CF10 3AT, UK; National Facility for X-ray Computed Tomography, Henry Royce Institute Hub Building, Manchester M13 9PL, UK; School of Chemistry, Cardiff University Main Building, Park Place, Cardiff CF10 3AT, UK; Wales Research and Diagnostic PET Imaging Centre, Cardiff University, University Hospital of Wales, Heath Park, Cardiff CF14 4XN, UK; School of Chemistry, Cardiff University Main Building, Park Place, Cardiff CF10 3AT, UK; Lancaster Environment Centre, Lancaster University, Lancaster LA1 4YQ, UK; North Central College, USA

**Keywords:** Agriculture, artificial intelligence, ecosystem management, micro-CT, mycorrhiza, plant health, positron emission tomography, quantum nanodot

## Abstract

Mycorrhizal fungi form essential symbiotic relationships with plant roots, facilitating nutrient exchange and promoting plant health. Understanding their interactions can benefit from advanced imaging techniques capable of visualizing nutrient exchange and structural colonization at subcellular resolution across large sample sizes. This review explores novel imaging approaches that are revolutionizing our understanding of root–mycorrhizal fungal symbioses. Several techniques can now visualize and characterize mycorrhizal fungi and associated root structures non-destructively and in three dimensions, for example X-ray computed tomography (micro-CT), X-ray fluorescence (XRF), and X-ray absorption near edge structure (XANES) spectroscopy. Metabolic processes and nutrient exchange can be tracked through positron emission tomography (PET), fluorescent nanoparticles (FNPs), and the monitoring of electrical signalling. Artificial intelligence (AI)-powered image processing software is enabling high-throughput analysis of complex images generated from a range of sources. Mycorrhiza systems are also able to be tracked in-field at multiple scales: hyperspectral imaging can detect mycorrhizal associations at the kilometre scale, while portable MRI imagers can detect changes at the tissue scale. These converging technologies enable the direct, continuous measurement of structural and metabolic root–mycorrhizal fungi interactions, paving the way for a mechanistic understanding of these vital symbiotic partnerships and their impact on plant health and ecosystem functioning.

## Teaser

Advanced characterization techniques (nanoSIMS, micro-CT, XRF, and XANES) and image analysis tools enable specific elemental and spatial mapping of mycorrhizal plant and fungal interactions and function *in situ*.

## Introduction

Mycorrhizal fungi are key mediators of ecosystem processes including interplant competition (Zhu *et al.*, 2023), nutrient cycling (Li *et al.*, 2023), and plant pathogen dynamics (Pujasatria *et al.*, 2024), and are key components of soil biodiversity required for achieving United Nations-mandated sustainable development goals on biodiversity, climate change, and food security (Pettorelli *et al.*, 2021). Understanding mycorrhizal function is key to their successful deployment and management. Specifically, fundamental biological questions remain regarding how these fungi spatially forage within the three-dimensional (3D) soil matrix, how carbon and mineral nutrients are dynamically traded at the root interface in real-time, how they operate under field conditions, and how hyphal networks respond physically to abiotic stress. However, resolving these dynamic, micro-scale processes presents unique methodological challenges (Martin and Van der Heijden, 2024).

Technological advances throughout history, such as light microscopy (Frank, 1891), electron microscopy (Scannerini and Bellando, 1968), culturing techniques (Mosse, 1956), and DNA analyses (Davison *et al.*, 2015; Chen *et al.*, 2018), have deepened our understanding of mycorrhiza function and distribution. Traditional tools, such as light and electron microscopy, have been foundational in characterizing static fungal morphology and quantifying overall root colonization. However, a drawback of these methods is that they are inherently destructive. Consequently, they often collapse complex 3D networks into 2D planes and restrict observations to isolated temporal snapshots under highly controlled conditions. This structural destruction fundamentally prevents the tracking of dynamic, *in vivo* physiological processes, such as continuous nutrient fluxes or progressive hyphal network expansion over time. Furthermore, *in situ* information about mycorrhizal interactions is limited as many current tools require the removal of mycorrhizal fungi from their environment for study. Thus, more research is needed to understand the influence of soil conditions on mycorrhizal fungal traits, function, and expression, and on the variance of mycorrhizal fungal traits with soil conditions (Lekberg and Helgason, 2018).

To address the limitation of traditional techniques, tools that preserve the spatial and temporal integrity of the symbiosis are required. By deploying advanced, non-destructive imaging techniques, the limitations of traditional approaches can be overcome. Techniques utilizing X-rays (Gahan *et al*., 2022; Keyes *et al*., 2022) and artificial intelligence (AI) (Jarratt-Barnham *et al*., 2024) allow for the preservation of 3D architecture, while radionucleotide (Komarov and Tai, 2022) and fluorescent tracers (Whiteside *et al*., 2009, 2012a, b; Hynson *et al*., 2015) enable the longitudinal tracking of metabolic exchanges *in vivo*. Innovations in forest-scale hyperspectral imaging (Sousa *et al*., 2021) and portable devices (Antonecchia *et al*., 2024; Blystone *et al*., 2024) also take this research from the laboratory into the field.

Here, we review how shifts from destructive, static sampling to continuous, 3D, field, and functional imaging is revolutionizing our understanding of root–mycorrhizal fungal interactions. We demonstrate using recent examples how advances in imaging are providing insights into the morphology of mycorrhizal fungi, their effects on root morphology, and their impacts for plant nutrient acquisition across diverse physical and chemical environments. The discussion extends from microscopic manipulations to field-based studies, highlighting the role of AI in automation and analysis. Finally, we conclude with a perspective on future directions and a comparative summary of these techniques (Table 1).

**Table 1.** A summary of AI image analysis relevant to mycorrhizal systems

Method name/Application area	Pros	Cons	Suitable application	Scale	References
Arbuscule characterization	Automates identification of arbuscules. Faster and more objective than manual methods.	May require training on specific datasets. Performance may vary depending on image quality.	Automating the identification and quantification of arbuscules in roots.	μm–mm	Evangelisti *et al*. (2021); Muta *et al*. (2022); Sciascia *et al*. (2023); Jarratt-Barnham *et al*. (2024)
Hyphal length and architecture	Semi-automated or automated measurement of hyphal length and other traits. Can handle complex images (HyLength, overlapping hyphae methods). RootPainter is adaptable.	Some tools require manual input (NeuronJ). Some tools may be limited by software availability (MATLAB). Some tools may overestimate thickness.	Automating the measurement of hyphal length, branching, and other architectural features.	μm–mm	Barry *et al*. (2015); Shen *et al*. (2016); Cardini *et al*. (2020); Smith *et al*. (2022); Sten *et al*. (2024)
Hyphal tracking and intracellular analysis	Track speed of intercellular particles.	ROC systems only.	Tracking hyphal growth and intracellular activity	μm–mm	Hammer *et al*., (2024); Oyarte Galvez *et al.* (2025)
Mycorrhizal maps	Provides spatial information.	Manual scoring component.	Visualizing colonization strategies.	μm–mm	Pop-Moldovan *et al*. (2022); Stoian *et al*. (2022)
Taxonomic classification	Can automate classification. Facilitates large-scale citizen science projects.	Performance can be limited by dataset size and variability. May not be applicable to all mycorrhizal types (e.g. AM fungi that are difficult to culture).	Automating the classification of mycorrhizal fungi (especially sporocarps and cultures).	mm–cm	Andrade *et al*. (2015); Melo *et al*. (2019); Picek *et al*. (2022); Kokkoris *et al*. (2024); Stiller *et al*. (2024); Struniawski *et al*. (2024)
Soil structure analysis	Can assess the impact of mycorrhiza on soil structure.	Destructive method.	Quantifying the effects of mycorrhiza on soil aggregation.	mm–cm	Jiménez-Martínez *et al*. (2024)

## Visualizing mycorrhizal and associated root structures

The architecture of a tissue is a fundamental driver of its biological function, serving as a critical metric for quantifying phenotypic plasticity and identifying specialized cellular structures. Furthermore, observing these structural patterns allows researchers to track developmental transformations and evaluate how a system specifically adapts or degrades in response to injury and disease (Pieruschka and Schurr, 2019; Acin-Albiac *et al.*, 2020). This connection between tissues and function also applies to root–microbe interactions (Birt *et al.*, 2022). Yet, the observation of mycorrhizal structures can be complex due to their microscopic nature and development in soil. Many widely utilized methods, for example analysis of extra-radical hyphal density and microscopic analyses of roots, provide limited functional understanding of the interactions between mycorrhizal fungi and their environment. Nevertheless, the range of tools available to overcome some of these difficulties is increasing.

**Fig. 1.**
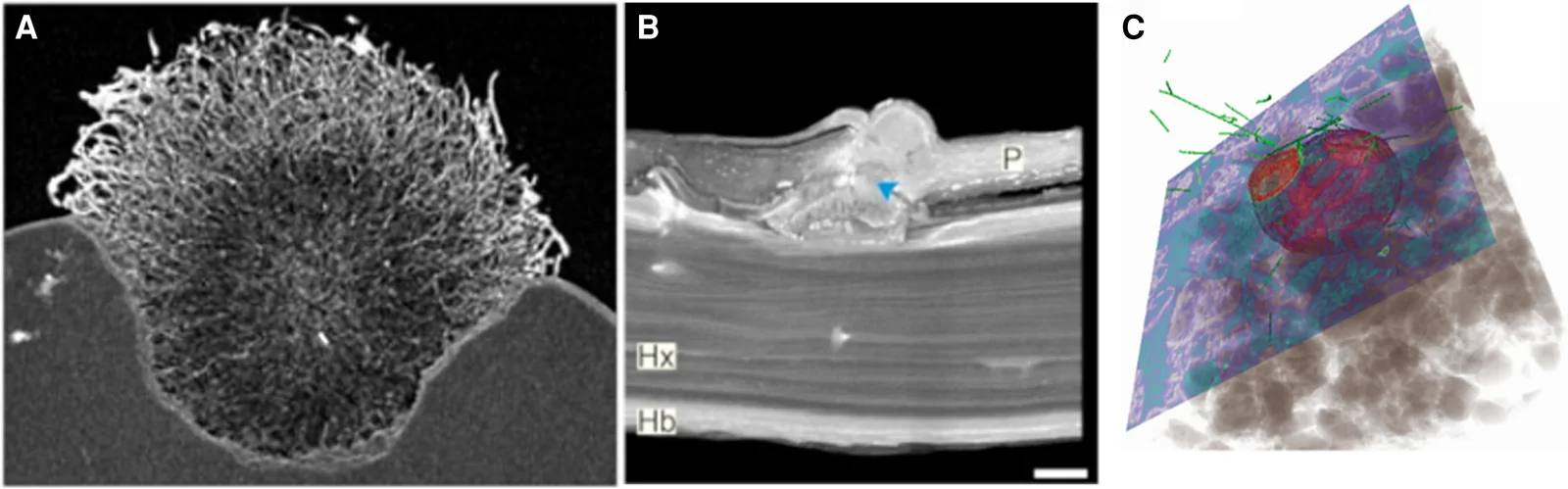
The application of micro-CT for imaging plants and fungi on the same scale as mycorrhiza. (A) *Aspergillus niger* external hyphal structure demonstrated by micro-CT (adapted with permission from Schmideder *et al.*, 2019). (B) Plant parasitic (*Struthanthus martianus*) structures demonstrated by micro-CT (adapted with permission from Teixeira-Costa, 2022): the parasite (P) structures, vascular strands, are clearly visible alongside host xylem (Hx) vessels, annual rings, and starch deposits within the host bark (Hb). Scale bar=1 cm. (C) *Rhizophagus irregularis* hyphae architecture (in green), exploring a soil aggregate, as demonstrated by micro-CT (adapted from Keyes *et al.*, 2022, CC-BY 4.0).

The limitations of 2D imaging can be overcome using X-ray computed tomography (CT). CT uses X-ray attenuation to generate cross-sectional images of a specimen, and micro-CT achieves this at an exceptionally fine resolution designed for small samples, typically in the range 0.5–20 cm^3^. Through rotational acquisition, numerous projections are reconstructed into a 3D model. The central advantage of micro-CT is that it is non-destructive, allowing internal microstructures of both mycorrhizal roots and fungi to be visualized and quantified *in situ* and in three dimensions. X-ray CT has been used to visualize plant root tissue (Mairhofer *et al.*, 2016; Piovesan *et al.*, 2021), fungi (Schmideder *et al.*, 2019; Fig. 1A), plant parasitic structures (Teixeira-Costa, 2022; Fig. 1B), and, more recently, arbuscular mycorrhizal (AM) fungal structures (Keyes *et al.*, 2022; Fig. 1C). Keyes *et al.* (2022) studied AM fungal hyphal topology through encouraging hyphae to grow from wheat roots into syringes filled with sand. This work found that the 3D hyphal morphology of the AM fungus *Rhizophagus irregularis* is not significantly dependent on phosphorus (P) availability (Keyes *et al.*, 2022). However, this application also highlighted a primary challenge of the technique: the highly radiopaque nature of the sand matrix obscured finer hyphal structures within smaller soil pores.

Several methodological adjustments have been explored to improve hyphal visibility against opaque substrates, but often these introduce trade-offs that undermine the benefits of micro-CT. For instance, we recently found that setting hyphae in agarose rather than sand improved imaging clarity due to its lower radiopacity (Video 1), but this approach required removing the hyphae from their initial growth medium (sand in this case), sacrificing vital *in situ* contextual information. Alternative X-ray-transparent media such as polymer foams, perlite, and glass beads may offer a compromise in that hyphae can potentially be imaged *in situ* and the substrates provide a degree of structural realism. However, some phytagels can melt under the high X-ray intensity used in some CT systems. Similarly, whilst submerging samples in contrast agents such as iodine increases tissue contrast at the cellular level (Wang *et al.*, 2017), introducing such chemicals may fundamentally alter biological systems.

Beyond substrate interference, achieving high-resolution scans often requires extended acquisition times, which means that radiation exposure becomes a secondary limitation. In plant systems, X-ray CT acquisition times >10 min have been shown to alter root growth patterns (Teramoto *et al.*, 2020). Careful consideration is therefore required regarding the effects of prolonged X-ray exposure on the development of mycorrhizal hyphae. To mitigate both scan time and resolution issues, researchers can utilize synchrotron X-ray sources (Keyes *et al.*, 2022). Synchrotrons generate incredibly high-intensity, low-divergence beams that drastically reduce imaging time while providing exceptional resolution and tuneable energy selection to optimize contrast.

Synchrotron sources can also be utilized in X-ray fluorescence (XRF) and X-ray absorption near edge structure (XANES) spectroscopy. XRF and XANES provide information on the composition of organic and inorganic substances. XRF provides information on the elemental composition of the sample, while XANES provides detailed information about the oxidation state, coordination environment, and electronic structure of specific elements, which are complementary when investigating structural composition (Gahan *et al*., 2022; Keyes *et al*., 2022). When coupled with detailed information on the anatomy of mycorrhizal fungal and plant tissue from micro-CT, both XRF and XANES have potential to provide exceptional insight into how structures are influenced at the microscopic scale. For example, Keyes *et al.* (2022) found that mycorrhizal fungal hyphae grow towards areas rich in organic sulfur, indicating a potential attraction to microbial communities. XANES is a spectroscopic technique that is highly sensitive to the oxidation state and local chemical environment of an element. By measuring the absorption of X-rays at and around the ‘K-edge’ (a specific energy where an electron is excited from a core shell), Keyes *et al.* (2022) obtained a unique ‘fingerprint’ for different chemical forms of sulfur.

One method that has been deployed to demonstrate functional interactions between mycorrhizal plants and fungi is nanoscale secondary ion MS (nanoSIMS). NanoSIMS is unique in that it can visualize and measure the distribution of elements and isotopes with nanoscale spatial resolution by irradiating the sample with a focused ion beam and analysing the ejected secondary ions using MS. The ultra-fine scale of isotope distribution afforded by nanoSIMS makes it possible to visualize nutrient distribution into some of the smallest mycorrhizal structures, such as arbuscules and fungal pelotons in orchids. Following ^13^C enrichment and sampling from an air gap between pot cultures, nanoSIMS confirmed that AM fungal hyphae are primarily constructed from carbon fixed by their plant host, as opposed to being scavenged from detritus (Kakouridis *et al.*, 2024). By investigating nutrient transfer in the opposite direction, nanoSIMS has also been deployed to reveal that the hyphae of fungi (identified only to class level, Hypocreales) can transfer nitrogen from ^15^N-enriched ant waste to *Caularthron bilamellatum*, an epiphytic orchid, in a mycorrhiza-like reciprocal nutrient exchange (Gegenbauer *et al.*, 2023).

Advanced imaging techniques, including micro-CT and positron emission tomography (PET), are revealing the structural and metabolic dynamics of beneficial root–fungus partnerships at sub-micron resolutions. Nevertheless, these techniques are often expensive, require specialized equipment and technical support, and often provide single-time snapshots. Electrical impedance tomography (EIT) is a non-invasive imaging technique that can provide *in situ* data about plant root architecture comparatively cheaply and with less specialized equipment. Combining EIT with manipulations of mycorrhizal fungi could offer novel information on the dynamics of mycorrhizal root and fungal interactions. For example, recent work shows that mycorrhizal fungi can influence plant root architecture and plant fitness (Liu *et al.*, 2022; Han *et al.*, 2023; Ain *et al.*, 2024), but these studies have been restricted to 2D imaging and a single time point. While EIT methods cannot directly detect the fine hyphae of mycorrhizal fungi, the method has been applied to other plant–fungal interactions. For example, EIT was used to show that *Brassica napus* infected with *Plasmodiophora brassicae* differed in its root phenotype compared with non-diseased plants (Corona-Lopez *et al.*, 2019). EIT has also been used to calculate the root biomass and surface area of a range of crops *in situ* (Michels *et al.*, 2024), providing important information on the temporal dynamics of root development.

Robotic microscopy is a promising tool for capturing the rapid, complex growth of mycorrhizal fungi. Oyarte Galvez *et al.* (2025) used a custom, high-throughput robot to monitor AM fungal networks continuously for 45 d. By moving a motorized camera assembly over a large area, the robot stitches together overlapping snapshots to create a 1.2 Gigapixel image of each fungal compartment at every time point. Using this technology, the researchers discovered that fungi grow in self-regulating ‘travelling waves’, where a moving front of nutrient-absorbing mycelium expands outwards. The density of this wave is controlled through fusion, a process that allows the fungus to expand efficiently with minimal carbon costs. This strategy enables the fungi to leave nutrient-poor zones and explore new areas for plant partners and resources, successfully balancing their energy trade-offs.

Acquiring longitudinal data is also achievable through more accessible and cost-effective approaches, and AMSlide is one such approach (McGaley et al., 2025). AMSlide is a 3D-printed sample preparation platform designed for non-invasive observations of plant root systems, intracellular mycorrhizal structures, and their interactions with the surrounding environment over time. AMSlide can be positioned in different ways, including on an inverted or upright microscope, and at an angle to guide roots containing mycorrhizal fungi that grow into the system downwards. Transmitted light can be used for imaging hyphae when grown in a clear substrate, such as agar and hydrogel (Paré *et al.*, 2022), and imaging is also possible in opaque media because roots grow against a transparent coverslip. External hyphae can be visualized using epi-illumination or autofluorescence, and removing opaque media from the platform enables imaging using both transmitted light and epifluorescence (McGaley *et al.*, 2025). Epifluorescence imaging is invaluable for studying soil fungi because it creates high contrast, allowing transparent hyphae to be clearly visualized against opaque soil particles that would otherwise obscure them in standard microscopy. By utilizing specific fluorescent dyes, this technique can distinguish fungal cell walls from other organic debris. Recent advances in label-free imaging could further enhance the AMSlide system, bypassing the need for dyes that are difficult to administer and may perturb the growth of fungal hyphae or the host plant. The use of pulsed near-infrared lasers has been shown to work well to take advantage of autofluorescence in plant–mycorrhizal fungi samples (Lee *et al.*, 2022); this could be used as a method for imaging in AMslide without dyes. Through using two-photon excitation fluorescence (TPEF) as a detection mode, this study was able to image the hyphae of mycorrhizal fungi in soil and plant roots without impeding their development and without the use of dyes.

## Tracking nutrient exchange and metabolic processes

Tracking how mycorrhizal fungi affect nutrient exchange and metabolic processes is key to understanding their impacts on plant health and broader ecosystem processes, and is often achieved using isotope labelling and imaging methods (e.g. Cameron *et al.*, 2006; Jacquemyn *et al.*, 2021). However, these approaches can sometimes restrict information on temporal dynamics of nutrient flows, or distribution of nutrients in 3D substrates, such as soil.

PET imaging can be applied to living organisms to image their interactions in three dimensions. This method makes use of positron-emitting radionuclides, which decay by emission of a positron that travels a short distance before an annihilation event with a nearby electron. This event produces a pair of γ-rays emitted in opposite directions that are detected by a cylindrical PET scanner. This coincident detection provides PET with exceptionally high sensitivity, detecting nano- or picomolar quantities of radionuclide. PET scanners often comprise an integrated CT scanner to provide parallel structural information. PET radionuclides can be incorporated directly in organic molecules (e.g. carbon-11 and fluorine-18) to produce radiotracers. For example, both 2-deoxy-[^18^F]fluoro-D-glucose and ^18^F-radiolabelled amino acids have been imaged in plants (Komarov and Tai, 2022). Metallic PET radionuclides can also be used, and these are typically chelated to a molecule of interest. By reconstructing the spatial distribution of these annihilation events, PET quantifies radiotracers, enabling 3D non-destructive visualization over time. PET has recently been used in plants to track fluorine metabolism (Tausta *et al.*, 2024), responses to drought (Miyoshi *et al.*, 2023b), carbon allocation to root nodules (Metzner *et al.*, 2022), and the translocation of sugars in response to transpiration (Miyoshi *et al.*, 2023a) in plants growing in both potting media and hydroponics. However, PET has rarely been used to study interactions between plants and mycorrhizal fungi, but it could be viable in the future for showing the movement of nutrients inside a hyphal network (Box 1). Extending these investigations to mycorrhizal symbioses would further enhance knowledge of plant systems to gain dynamic, spatially explicit data on carbon and mineral nutrient allocation, and responses to abiotic stress (Geisler *et al.*, 2023; Lekberg *et al.*, 2024). For example, the ability to track lipids would be particularly timely following the discovery that AM fungi are fatty acid auxotrophs and rely on plant-derived lipids (Jiang *et al.*, 2017; Keymer *et al.*, 2017; Luginbuehl *et al.*, 2017; Box 1).

Box 1. Case study one: expanding the application of PET for mycorrhizal image analysis

**Fig. 2.**
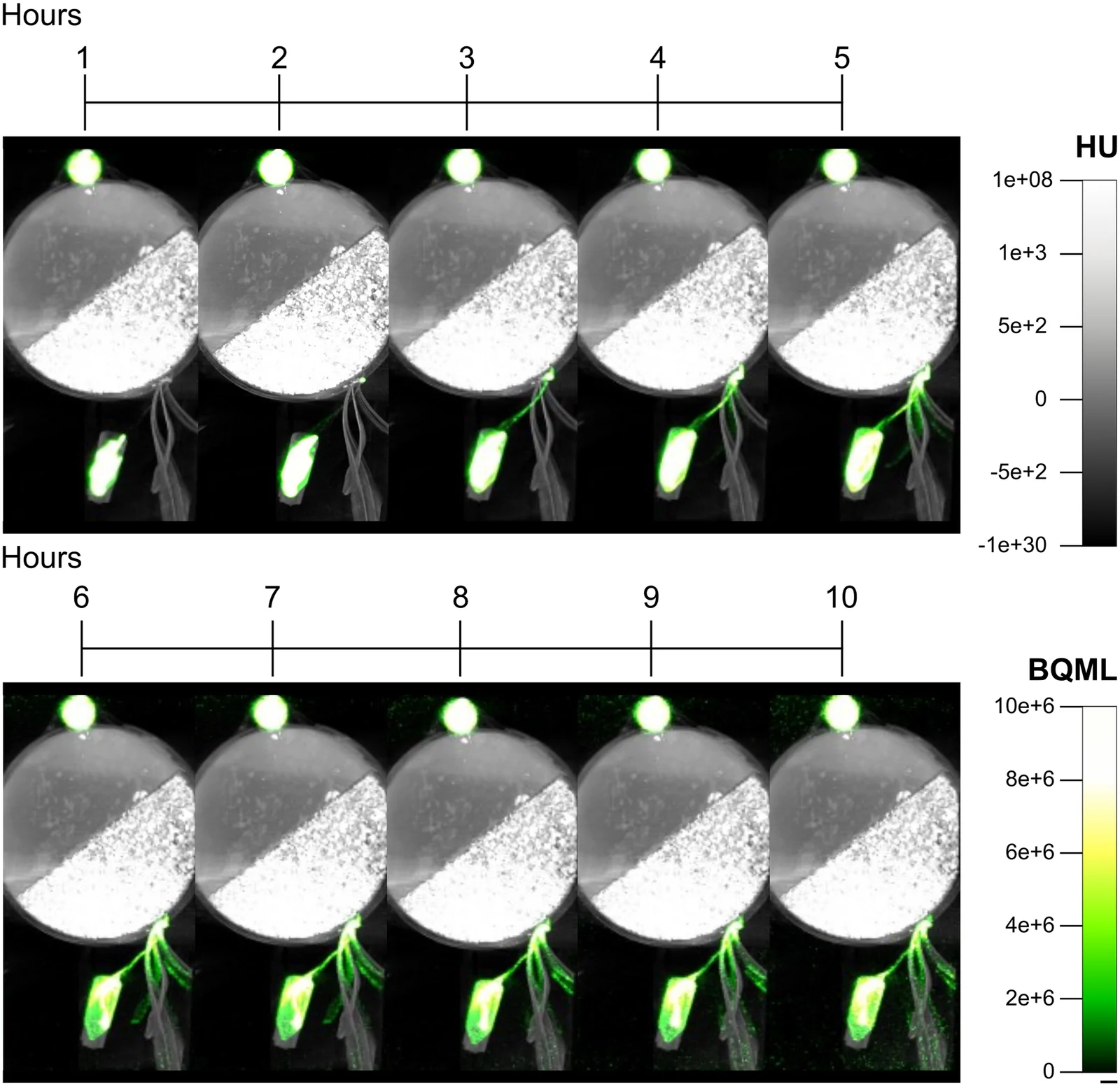
Maximum intensity projection (MIP) views of a PET/CT hourly time course of the uptake of 11-[^18^F]fluoroundecanoic acid by a leaf of *Plantago lanceolata* immersed in a solution of the tracer. The use of PET-labelled fatty acid substrates has been demonstrated elsewhere (Cai *et al.*, 2016). No uptake was observed via the root, which was also dipped in a separate vial containing 20 MBq of the same tracer (top of image).

PET has potential to enhance our understanding of mycorrhizal function, for example through tracking lipid movement in plants using a fluorine-18 radiolabelled analogue of undecanoic acid or other similar fatty acid. The tracking of fatty acids using PET-labelled substrates has been demonstrated in previous work (Cai *et al.*, 2016), and the example below (Fig. 2) shows a leaf of *Plantago lanceolata* immersed in synthesized 11-[^18^F]fluoroundecanoic acid. In this example, the [^18^F]fatty acid could be imaged for up to 10 h. This method could be utilized to image key plant tissue in commercially available pre-clinical PET/CT scanners (e.g. Mediso Nanoscan 122S) that are widely used in the medical field. This approach has the benefit of providing a 3D image as well as a time series (Video 2). Nevertheless, there is a need to carefully select radionuclides according to the investigation being undertaken. For example, the rate of movement of fatty acids through plant tissue may require the use of longer-lived radionuclides, whereas other ecologically important processes, such as sugar transport, are better suited using ^18^F which has a half-life of 109.7 min (Miyoshi *et al.*, 2023a).

Previous experiments tracking fatty acids in the plant–mycorrhizal fungi symbiosis have incubated labelled substrates for weeks (Jiang *et al.*, 2017; Keymer *et al.*, 2017), and evidence from plant systems in the absence of mycorrhizal fungi suggests that this may be the amount of time required to translocate fatty acids from the leaves to roots (Terzaghi, 1989). Therefore, novel radionuclides may need to be utilized to image plant–mycorrhizal fungi symbiosis effectively using PET, and longer-lived radionuclides such as ^89^Zr and ^48^V may be useful in this respect. The 78.4 h half-life of ^89^Zr, which allows for ∼15 d of imaging from a single injection of radiolabelled agent, is well suited for imaging slowly circulating biologically derived macromolecules and agents. As a result, ^89^Zr is now a well-established radioisotope in the labelling and pre-clinical imaging of cells and antibodies, although its potential has yet to be explored in plant imaging. Also of promise for plant imaging is the isotope, ^48^V, which has a 15.97 d half-life and potentially allows for up to 2.5 months of imaging from a single administration. ^48^V has a considerably larger atomic radius than carbon, reducing its utility for the study of fatty acids, but is a close chemical analogue of phosphorus (Olness *et al.*, 2001). The use of these novel radionuclides requires highly specialized cyclotron facilities for their production (Broder *et al.*, 2022), which are often primarily used by medical researchers, and so there may be logistical constraints that limit their use by natural scientists. Similarly, medical scanners currently used in PET often have a horizontal bed and may lack sufficient climate control to manipulate plant systems. However, there are multiple projects underway to develop PET scanners specifically for plant systems that may overcome these issues in the future (Hinz *et al.*, 2024; Yamamoto *et al.*, 2024), and the general shift in funding for cross-disciplinary research projects in many countries may also drive innovation in this area.

Fluorescent nanoparticles (FNPs) have been used to track nutrient exchange between plants and mycorrhizal fungi (Whiteside *et al.*, 2009). FNPs are extremely small clusters of organic or inorganic materials with a crystalline or non-crystalline structure (Färkkilä *et al.*, 2021), and, when exposed to light, they emit specific wavelengths causing them to fluoresce. FNPs can be added to a biological system through being attached to a molecule of interest, and are typically long-lived, bright, and have tuneable fluorescence. These properties are due to their fluorescence wavelength being related to their size and surface properties (reviewed in detail in Färkkilä *et al*., 2021).

FNPs have been used to demonstrate that AM fungi directly take up amino acids from the environment, and that they can have specific preferences depending on environmental conditions, notably N fertilization (Whiteside *et al.*, 2009, 2012a, b; Hynson *et al.*, 2015). The ability to simultaneously tag 20 different amino acids using FNPs was key to facilitating these discoveries. More recently, FNPs were used to show that mycorrhizal fungi networks transferred P in response to host demand and environmental stress (Whiteside *et al.*, 2019; Van’T Padje *et al.*, 2020a, b, 2021). FNPs were also used to demonstrate that including more distantly related mycorrhizal fungi in a network increases the size of the network but not the benefit to the plant host (Van’T Padje *et al.*, 2022). Despite the advances in understanding which these studies have provided, concerns have been raised about the toxicity of FNPs, particularly those containing cadmium (Sobhanan *et al.*, 2023). The manufacture of FNPs can have drastic impacts on their final properties, including heavy metal toxicity, that could affect the plant and fungi being studied, underscoring the need for carefully designed and appropriately matched controls to account for such effects (Yang *et al.*, 2023). Further study into how different classes of FNPs can influence mycorrhizal fungi will facilitate their effective use in future research.

Measuring electrical signals offers another promising avenue for tracking metabolic processes. Electrical signals function as rapid, long-distance communication networks to coordinate physiological responses to environmental stimuli, such as stress, resource availability, and damage. This phenomenon is well established for plants and has been proposed to occur to explain functioning of common mycorrhizal networks (Johnson and Gilbert, 2015). A better understanding of electrical signalling could allow the role of mycorrhizal fungi to be exploited more widely, including as sentinel plants in crop production systems (Pickett and Khan, 2016; Alaux *et al*., 2021). Beyond pest and disease management, mycorrhizal fungi also mediate responses to drought (Zhang *et al.*, 2024) and light availability (Fellbaum *et al.*, 2014), and these stimuli can also cause plant electrophysiological responses (Pachú *et al.*, 2023; Tian *et al.*, 2023; Li *et al.*, 2024). Therefore, the advances of electrophysiology in plants could be extended to mycorrhizal fungi to track their role in these plant metabolic processes.

Utilizing electrode-based approaches to monitor these signals could address fundamental questions about root–mycorrhizal interactions, such as determining the rate at which stress signals are propagated across a mycelial network, assessing whether electrical signalling coordinates nutrient exchange at the root–fungus interface, and observing how the symbiotic network responds in real time to external abiotic and biotic factors. The merits of this theory and further information on likely fungal physiological processes associated with electrical signalling have been extensively reviewed (Buffi *et al.*, 2025), but here we discuss them with respect to mycorrhizal fungi. Despite a report of AM fungi mycelium transmitting electrical signals (Thomas and Cooper, 2022), other authors questioned a lack of controls as well as providing suggestions for experimental designs (Blatt *et al.*, 2023, 2024). Voltage-sensitive dyes are one way in which plant electrical signals have been measured without conventional extracellular or intercellular probes (Qin *et al.*, 2013). These dyes change their spectral properties in response to voltage and have shown that the phloem is the main conduit for plant electrical signals within leaves (Zhao *et al.*, 2015). It would be interesting to test whether these signals continue to roots and mycorrhizal fungi, although consideration of the toxicity of the dyes is required (Qin *et al.*, 2013; Zhao *et al.*, 2015).

Genetically encoded voltage indicators (GEVIs) are a non-invasive method that may overcome this constraint. GEVIs are proteins that fluoresce in response to changes in membrane potential and have been shown to be effective in fungal (Limapichat, 2022) and plant cells (Reyer *et al.*, 2020), but their application to AM fungi may be limited because of the inability to genetically transform the fungi. This constraint means that GEVIs are not feasible for visualizing electrical signals directly within AM fungal networks, though they remain relevant for studying the plant side of the symbiosis or other transformable mycorrhizal fungal species. However, more conventional microelectrode recordings can also be integrated with fluorescent probes. This approach has revealed that action potentials triggered by hydrogen peroxide also set off a wave of calcium ions within the plant cell (Koselski *et al.*, 2023).

## Field applications of mycorrhizal imaging

It is widely acknowledged that many critical features of mycorrhizal symbioses may differ significantly between controlled laboratory and field conditions (Lekberg and Helgason, 2018), and so it is important to validate laboratory findings *in situ* wherever possible. Hyperspectral imaging is one approach that is furthering the understanding of mycorrhizal associations in the field. Hyperspectral imaging analyses plant leaves by capturing light across hundreds of narrow spectral bands, extending far beyond the red, green, and blue range of human vision. This detailed spectral signature enables the detection of subtle biochemical changes, such as fluctuations in chlorophyll, water content, and nitrogen concentrations. Consequently, it captures non-destructive information related to plant stress, disease, and nutrient deficiencies before visible symptoms appear on the leaf surface. Hyperspectral satellite data of tree canopies can predict whether trees associate with either ectomycorrhizal (ECM) or AM fungi, because AM canopies absorb more light in wavelengths related to biochemical properties of leaves compared with ECM canopies (Sousa *et al.*, 2021). This study suggested that using hyperspectral imaging worldwide could significantly enhance the accuracy of mapping distributions of mycorrhizal associations, increasing the precision of current global mycorrhizal maps by ∼10 000 times. Furthermore, it would enable the integration of dynamic changes in forest mycorrhizal associations into global models, leading to more accurate predictions of ecosystem responses to environmental change. Hyperspectral imaging has also been applied to assess the impact of AM fungal inoculation on plant P acquisition and responses to drought in greenhouse conditions (Kong *et al.*, 2020; Sun *et al.*, 2021). Accurate assessment of P and water status in AM fungal-inoculated crops can lead to improved crop management strategies, maximizing the benefits of AM fungal inoculation while minimizing environmental impacts associated with excessive P fertilization or wasteful irrigation. Together, these methods could make the assessment of large numbers of mycorrhizal plants at the landscape scale possible, thus advancing our understanding of how mycorrhizal associations correlate across climatic conditions and soil conditions.

Imaging mycorrhizal associations in the field requires portable equipment; portable PET scanners for the assessment of plant stress in the field are currently under development (Antonecchia *et al.*, 2024). Portable MRI machines for plant science are also being developed which offer another method of assessing mycorrhizal interactions non-destructively in the field (Blystone *et al.*, 2024). MRI works by using a powerful magnetic field to align the hydrogen protons in target tissues and then disturbs that alignment with radiofrequency pulses to detect the unique energy signals emitted as the protons return to their original state. Portable MRIs can image a range of parameters such as plant water content, xylem and phloem flow, root topology, physiological changes, and disease progression, and these attributes can change in response to mycorrhizal association, making them useful targets for assessment in the field (Veresoglou and Rillig, 2012; Zhang *et al.*, 2024).

Portable root imaging cameras, such as the CI-602 narrow gauge root imager (CID BioScience, USA), have potential to provide longitudinal image data in the field. The CI-602 functions by inserting a high-resolution, cylindrical scanner into pre-installed, transparent acrylic tubes buried in the soil. Once inside, the camera head rotates to capture a 360° digital image of the roots and soil profile without disturbing plant growth. These high-resolution scans enable analysis of root development, turnover, and architecture over time non-destructively. For example, root imagers in combination with traditional microscopy were used to characterize ECM fungal morphotypes and monitor their development seasonally in different forest types (Thiem *et al.*, 2023). As well as elucidating the ability of various ECM fungi to endure a range of soil salinities, this study emphasizes the benefit of combining novel mobile imaging and traditional microscopy techniques to address specific hypotheses.

## Emerging technologies in the manipulation of mycorrhizal systems for imaging

**Fig. 3.**
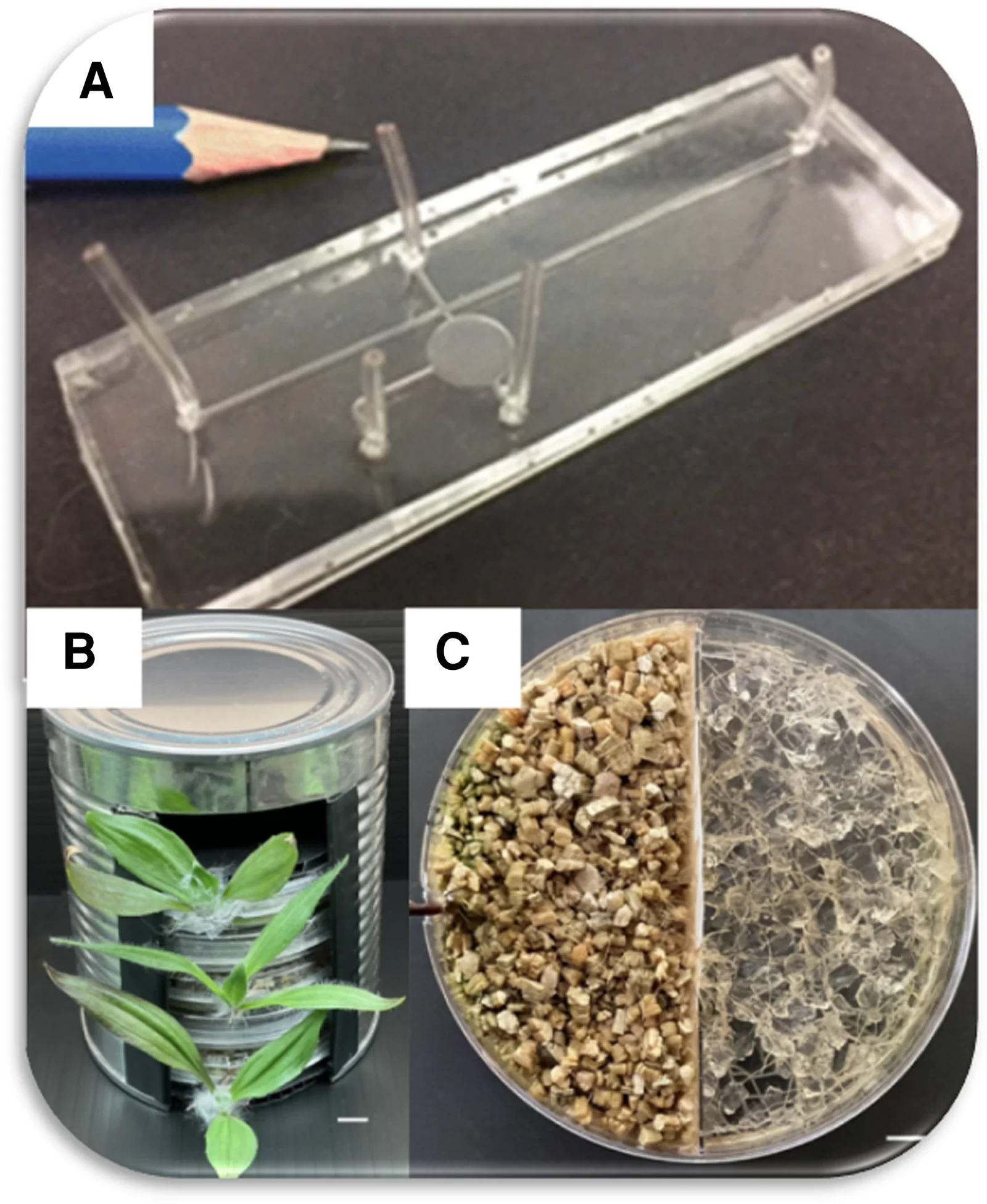
Emerging technologies for the manipulation of mycorrhizal systems. (A) A microfluidic chip used for mycorrhizal spore separation (adapted with permission from Srisom *et al.*, 2020). (B) *Plantago lanceolata* growing from a SAP. (C) the mycorrhizal SAP section shown alongside the vermiculite plant section that is separated by a mesh (adapted from Paré *et al.*, 2022, CC-BY 4.0).

Understanding causal relationships in mycorrhizal systems requires tools that permit precise manipulation of fungal growth, nutrient gradients, and spatial configuration under live-imaging conditions. Microfluidic devices (Fig. 3A) fulfil these requirements by enabling controlled microscale environments compatible with high-resolution imaging. Combining manipulative systems with novel imaging techniques allows researchers to isolate specific effects, visualize dynamic processes, and study spatial relationships.

Microfluidic devices can manipulate and control very small amounts of fluids (1 nl to 1 pl), typically in channels with dimensions <1 mm. These minute channels can be combined with imaging techniques to explore how hyphal growth and branching respond to different environments. For example, *Rhizophagus irregularis* grown in microfluidic chips were shown to prefer straight rather than convoluted paths to forage (Hammer *et al.*, 2024). Furthermore, microfluidics may also shape phenotyping of mycorrhizal fungi. For instance, Richter *et al.* (2024) explored hyphal foraging strategies of *Rhizophagus* spp. and *Gigaspora margarita* on a microfluidic device and found that each species exhibited distinct germination, growth, and branching patterns. The hyphae of the *R. irregularis* strain could retract its cytoplasm and then reverse this process by forming and breaching septa. Microfluidic systems enable these types of studies because they not only facilitate tight control over abiotic parameters such as nutrient concentrations, pH, and water availability, but also allow real-time observations and can be scaled to provide high-throughput systems (Takahashi *et al.*, 2023). Nevertheless, current microfluidic systems represent a simplified model compared with fluidic channels found in a real soil (Zhang *et al.*, 2022). Future designs will need to mimic soils to provide accurate information about how mycorrhizal fungi would act in their actual environment.

The development of transparent media that have properties akin to soil offers promise for generating ecologically relevant images of mycorrhizal fungi (Klein *et al.*, 2024). Other media such as agar may produce a non-ecologically relevant network topology, and the true form produced in soil is difficult to image using current methods. One transparent medium uses a superabsorbent polyacrylate polymer (SAP) for the non-sterile cultivation of AM fungi while maintaining a plant host (Paré *et al.*, 2022; Fig. 3B, C). This system has several advantages over pot culture or root organ cultures (ROCs), such as the simplicity in their establishment and ability to be maintained in non-sterile conditions. Crucially for image acquisition, the refractive index of the media is similar to that of water, making the imaging of hyphae easier than in opaque soil matrices. Using this system, it has been demonstrated that *R. irregularis* can form dimorphic spores that are miscategorized as *R. fasciculatus* (Kokkoris et al., 2024). Through utilizing SAP, the spore dimorphism could be demonstrated to occur in non-sterile conditions and the hyphae could be tracked as they grew.

## Artificial intelligence and automation in image analysis

Traditionally, quantifying the colonization of roots and substrates by mycorrhizal fungi has primarily relied on manual microscopy methods. However, such methods are often time-consuming, labour-intensive, and prone to biases (Corona Ramírez *et al.*, 2023). To address these bottlenecks, digital microscopy and image analysis techniques offer faster, more accurate, and objective assessments.

To streamline the analysis of key metrics such as hyphal density and architecture, semi-automated digital tools were initially developed. One of the first automated image tools designed to track hyphal growth, length in extracted samples, and hyphal branching topology was AnaMorf, developed as a plug-in for the popular image processing software ImageJ (Barry *et al*., 2015). This tool was used initially to track the development of the hyphae and spores of *Aspergillus oryzae* on agar, but has since had limited use in mycorrhizal studies. Another ImageJ plug-in, used for tracking neuron development, NeuronJ, has been applied successfully to images of AM fungal hyphae (Shen *et al.*, 2016), demonstrating that mycorrhizal fungal hyphae proliferate in response to low P availability. However, NeuronJ requires semi-manual tracing of hyphae, limiting its applications due to the amount of time required to process images. More recently, the HyLength tool was developed as another semi-automatic plugin for ImageJ that performed better than AnaMorf in terms of measuring time and ability to deal with complex images (Cardini *et al.*, 2020). Yet, this approach has been improved further still by considering overlapping hyphae in 2D images from 3D structures (Sten *et al.*, 2024). Nevertheless, the implementation of this method in the proprietary software MATLAB may limit its widespread adoption.

Beyond structural architecture, researchers have also utilized these foundational tools to study cellular contents. Using microfluidics and image analysis tools such as ImageJ, it is possible to automatically track the speed and direction of intracellular particles within hyphae as opposed to having a researcher track manually through videos or images. This approach, using ROCs, revealed that cytoplasm velocity is highest at the tip of AM fungal hyphae, decreases as it moves towards the plant, and moves in pulses (Hammer *et al.*, 2024). Further investigation is needed to determine if these cytoplasmic velocities determined in ROCs differ in mycorrhizal fungi associated with fully functional plants.

While semi-automated tools offer reliable workflows and easy access to a suite of traits, they present significant limitations: they usually require a highly controlled, specialized setup with low background noise to function, or they involve time-consuming manual tracing of relevant tissue structures. To overcome these limitations, recent advancements have increasingly deployed deep neural networks. A primary advantage of deep learning algorithms, such as convolutional neural networks (CNNs), is their capacity to be trained to recognize an array of shapes and separate them from complex background noise (e.g. soil particles, bubbles, droplets, or staining artefacts).

New deep-learning tools allow researchers to train underlying CNNs and tailor target detection to specific, messy environmental samples. For example, new tools such as RootPainter (Smith *et al.*, 2022), which was originally developed to find roots in rhizotron images, allow researchers to train the underlying CNN and tailor its detection to specific use cases. This software has been successfully employed to detect roots, soil biopores, root nodules, and enchytraeids in soil (Smith *et al.*, 2022; Van Hall and Van Gestel, 2025). Once the model can recognize target structures, follow-up software can extract relevant features. For example, the CNN may be trained to detect hyphae in extracts from soil or other media, followed by extraction of hyphal length, average diameter, or even branching patterns by tools such as WinRHIZO (Regent Instrument Inc., Canada) or RhizoVision (Seethepalli *et al.*, 2020). The CNN could be trained to find other mycorrhizal structures, such as vesicles, which are then counted by ImageJ (Schneider *et al.*, 2012). Therefore, the use of customized image recognition, trained on the specific characteristics of their images, opens up several possibilities for future projects. We provide an illustrative example of what the output from using CNNs could look like (Box 2). Further investigation is required to assess whether they could be a viable option for automation in image analysis for studies of mycorrhizal fungi.

Box 2. Case study two: customized image recognition to assess hyphal traits

**Fig. 4.**
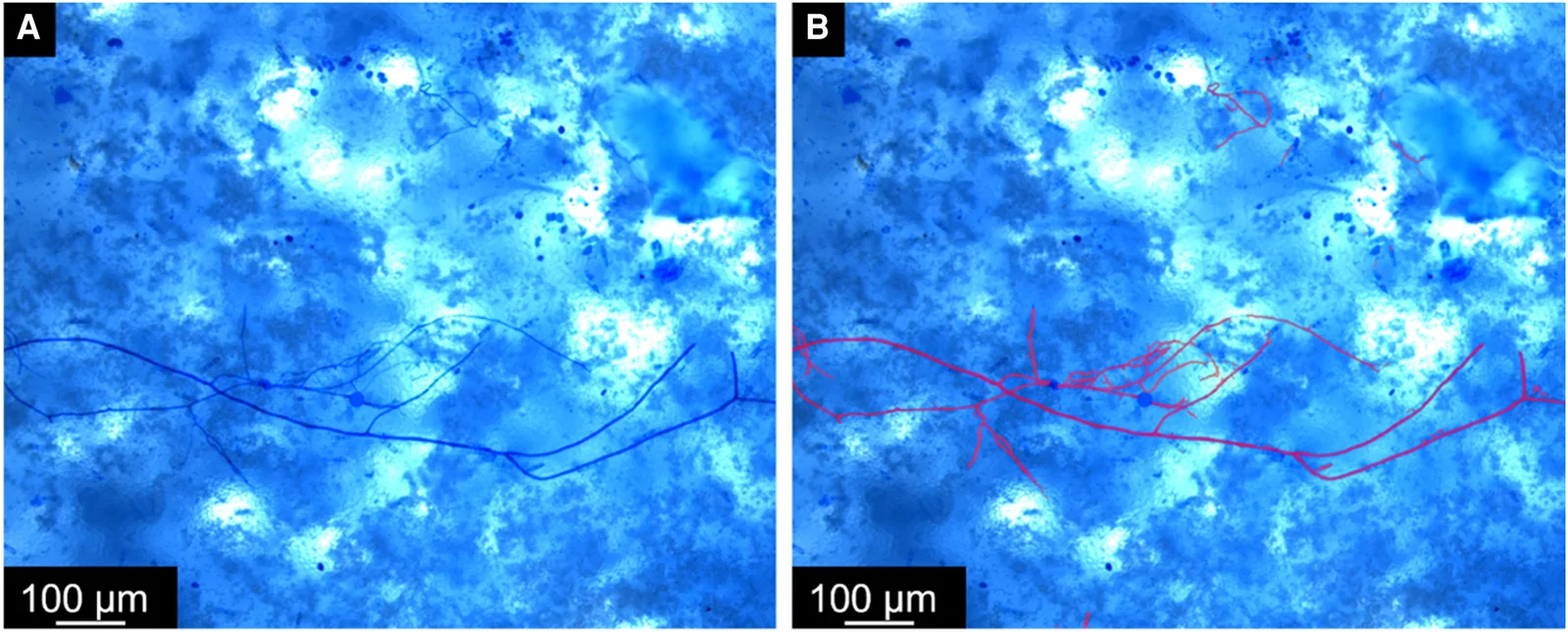
Microscopic image comparison demonstrating machine learning-based segmentation of fungal hyphae embedded within a hydrogel matrix. (A) Microscopic image of a hydrogel extract containing hyphae, stained with blue ink. (B) The interactively trained convolutional neural network (RootPainter) can recognize hyphae (pink) among the high abundance of complex structures with similar colour and contrast to the hyphae (Smith *et al.*, 2022). It was also successfully trained to exclude attached and detached vesicles or spores, but it overestimated the thickness of the finest hyphae. Original image but similar approaches have been used by Stiller *et al.* (2024).

RootPainter (Smith *et al.*, 2022) is a tool that could be used to recognize the hyphae of mycorrhizal fungi (Fig. 4A, B). Unlike other tools, RootPainter is trained interactively by visually directing the model to target features and correct misidentifications during training, for which a user interface is provided. The setup and training can be achieved via cloud computing (Google Colab) and can therefore be performed on standard computers. For the training to be successful, the target structure must be clearly defined, as the more complex the target is, the more training data are required (Fig. 4). The resulting images can easily be analysed using WinRHIZO or RhizoVision to acquire information on hyphal length, diameter distributions, and branching patterns. Importantly, the enormous number of images that can be processed with such a tool suggests promising future applications, for example investigating the relationship between hyphal traits and ecosystem processes.

One of the areas of analysis of mycorrhizal systems where the application of automatic image analysis has been widespread is the characterization and quantification of arbuscules in roots. AMFinder (Automatic Mycorrhiza Finder) is a software tool designed using CNNs to automate the identification and quantification of fungal colonization in stained plant roots (Evangelisti *et al.*, 2021). Other methods have utilized similar machine learning protocols with equal success to recognize arbuscules (Muta *et al.*, 2022; Sciascia *et al.*, 2023). Recently, AMFinder was integrated twith rained models into an R package (AMReader) which potentially gives better performance on poorer images (Jarratt-Barnham *et al.*, 2024).

Machine learning applications have also been applied in mycorrhizal systems beyond subcellular contents. For example, vision transformers decompose an image into a sequence of discrete patches, processing them like words in a sentence, and identifying global relationships between distant parts of an image. This model facilitated a large citizen science project to vastly increase a public dataset, the Atlas of Danish Fungi (Picek *et al*., 2022). Another recent machine learning approach used a CNN algorithm to classify filamentous fungi, including those that are potentially mycorrhizal, such as the Mucoromycota (Stiller *et al.*, 2024; Fig. 4). Although the model showed poor performance at lower phylogenetic ranks (an accuracy of <49% below order), it was limited by the small dataset size, uneven class distribution, and the inherent variability in the appearance of individual fungal colonies from the same species. Nevertheless, the findings suggested that visual characteristics such as colour, patchiness, and growth rate are useful for identifying fungi at broader taxonomic levels of phylum, class, and order. This approach focused on sporocarps and cultures, preventing the analysis of AM fungi, although it has been shown that machine learning algorithms can classify pathogenic fungal spores from four different genera with relatively high accuracy (82%) (Struniawski *et al.*, 2024). Although these models have yet to be applied to AM fungal spores, they could be particularly effective given that spore morphology is one of the traditional methods for species classification of AM fungi (Kokkoris *et al.*, 2024). While yet to be used for taxonomy, machine learning algorithms have also been successfully applied to automate spore counting (Andrade *et al.*, 2015; Melo *et al.*, 2019).

As these automated tools generate an unprecedented volume of data, new methods are required to synthesize this information into ecologically meaningful formats. Mycorrhizal maps are digital visualizations (obtained by capturing images of, for example, stained roots) that chart the exact spatial distribution of, for example, hyphae, arbuscules, and vesicles within the architecture of a plant root (Pop-Moldovan *et al.*, 2022; Stoian *et al.*, 2022). Pop-Moldovan *et al.* (2022) utilized these maps to demonstrate that an amino acid-based biostimulator acts as a ‘slow-motion inducer’, altering fungal strategies from active nutrient transfer to restricted expansion and storage across different fungal growth stages. This image analysis technique may prove particularly useful when combined with the large volumes of data generated with automated recognition of fungal structures within images.

## Conclusion

We suggest the following areas of priority research.

First, exploiting advanced imaging techniques of mycorrhizal systems to better understand functional and trait variability. Recent research highlights significant intraspecific genetic variation within AM fungi (Chen *et al.*, 2018) and multiple lineages where ECM fungal associations evolved (Looney *et al.*, 2022). As such, focusing on the traits of mycorrhizal fungi and phenotypes, rather than taxonomy, will improve our understanding of their ecological impact (Chaudhary *et al.*, 2022).

Second, capturing function and structure in 3D and opaque substrates. Comparatively little is known about mycorrhizal fungal foraging strategies and interactions in soil. While CT methods have been shown to contribute to furthering this understanding (Keyes *et al.*, 2022), there is a need to expand the application of 3D imaging. This expansion should be in terms of both the availability of equipment to researchers and the number of samples that can be processed. Continuing interdisciplinary collaboration will provide further opportunities to deploy 3D imaging more widely; although better image processing may be able to determine more 3D information from 2D images in the near-term (Sten *et al.*, 2024).

Third, continuing to understand how mycorrhizal fungi operate in the field (Lekberg and Helgason, 2018). Portable imaging such as mobile PET or MRI scanners offers promise (Antonecchia *et al.*, 2024; Blystone *et al.*, 2024), but exploring other approaches such as using satellite data will further advance this goal.

Fourth, expanding the use of high-resolution manipulations of mycorrhizal environments. For example, the use of microfluidic chips offers valuable insights into how mycorrhizal fungi respond to their environment (Hammer *et al.*, 2024). Expanding the use of these systems into more realistic settings, including the move away from ROC to full plants and further aiming to replicate the complexities of soil, will increase the ecological relevance of their findings.

Fifth, increased automation especially of image analysis. Automation for tracking mycorrhizal structure (Barry *et al*., 2015; Shen *et al*., 2016; Sten *et al*., 2024) and metabolic processes (Hammer *et al*., 2024) will be vital to converting images into quantitative data for analysis. Continuing to develop novel analysis tools such as mycorrhizal maps (Pop-Moldovan *et al*., 2022; Stoian *et al*., 2022) will also be important to garner insights into mycorrhizal ecology.

Sixth, better integration of approaches. Addressing the most significant methodological challenges will probably require the convergence of the imaging technologies described in this review (summarized in Table 2) with the plethora of other techniques already available. Such approaches require interdisciplinary thinking and practice but promise to unlock a deeper understanding of mycorrhizal interactions.

**Table 2.** A summary of emerging imaging technologies relevant to mycorrhizal systems

Method name/Application area	Method category	Pros	Cons	Suitable application	Scale	References
X-ray computed tomography (micro-CT)	Visualizing mycorrhizal fungi and associated root structures	Non-destructive. 3D visualization of root architecture and fungal colonization. High resolution. Can be combined with XRF and XANES for elemental/chemical composition.	Can be slow (lab-based systems). Prolonged X-ray exposure can affect growth. Radiopaque media can obscure structures. Expensive equipment and specialized support.	Visualizing and quantifying root and mycorrhizal structures in 3D. Studying hyphal morphology and response to soil conditions.	μm	Walker and Powell (1979); Mairhofer *et al*. (2016); Wang *et al*. (2017); Teramoto *et al*. (2020); Piovesan *et al*. (2021); Keyes *et al*. (2022)
Synchrotron X-ray techniques (including XRF and XANES)	Visualizing mycorrhizal fungi and associated root structures	Very fast image acquisition. Exceptional resolution. Tuneable energy for optimized contrast. XRF: elemental composition. XANES: oxidation state, coordination environment, and electronic structure of elements.	Limited access to synchrotron facilities. Expensive. Single time-point snapshots. Less information on the associated root.	Focused on detailed analysis of hyphal structure and interaction with soil chemistry. Investigating elemental distribution and chemical transformations.	μm	Taylor *et al*. (2009); Gahan *et al*. (2022); Keyes *et al*. (2022); Schütz *et al*. (2022)
Nanoscale secondary ion mass spectrometry (nanoSIMS)	Visualizing mycorrhizal fungi and associated root structures	Extremely high spatial resolution (nanoscale). Visualizes and measures element/isotope distribution.	Expensive. Requires specialized equipment and support. Single time point snapshots.	Tracing nutrient transfer (e.g. carbon, nitrogen) between plants and fungi. With a focus for how these may accumulate in either fungal or plant tissue.	μm	Gegenbauer *et al*. (2023); Kakouridis *et al*. (2024)
AMSlide	Visualizing mycorrhizal fungi and associated root structures	Non-invasive observation of root systems and interactions. Can be used with transmitted light (clear media) or epifluorescence (opaque media). Relatively inexpensive and simple.	Limited to 2D imaging with transmitted light. Requires specific media (clear for transmitted light).	Observing dynamic processes such as arbuscule formation over time. Studying hyphal growth in different media.	μm–mm	Paré *et al*. (2022); McGaley *et al*. (2025)
Label-free imaging (TPEF)	Visualizing mycorrhizal fungi and associated root structures	Visualizes hyphae in soil without labelling.	Limited examples of use for troubleshooting.	Studying mycorrhizal hyphae in soil and plant roots.	mm	Lee *et al*. (2022)
Fluorescent nanoparticles (FNPs)	Tracking nutrient exchange and metabolic processes	Bright and long-lasting fluorescence. Tuneable fluorescence based on size/surface properties. Can be used to tag multiple molecules simultaneously.	Potential toxicity (especially cadmium-containing FNPs). May negatively influence microbes.	Tracking nutrient uptake and movement (e.g. amino acids, phosphorus) within mycorrhizal networks.	μm–mm	Whiteside *et al*. (2009, 2012a, b, 2019); Hynson *et al*. (2015); Van’T Padje *et al*. (2020a, b, 2021, 2022); Färkkilä *et al*. (2021); Sobhanan *et al*. (2023); Yang *et al*. (2023)
Plant electrical signals (with voltage-sensitive dyes, GEVIs, or microelectrodes)	Tracking nutrient exchange and metabolic processes	Continuous measurements over time. Can provide insights into communication and signalling.	Voltage-sensitive dyes can be phytotoxic. GEVIs require genetic modification. Direct measurement of mycorrhizal signals is challenging.	Investigating the role of electrical signals in mycorrhizal communication and responses to stimuli.	μm–mm	Babikova *et al*. (2013); Qin *et al*. (2013); Fellbaum *et al*. (2014); Johnson and Gilbert (2015); Zhao *et al*. (2015); Pickett and Khan (2016); Reyer *et al*. (2020); Limapichat (2022); Thomas and Cooper (2022); Blatt *et al*. (2023, 2024); Koselski *et al*. (2023); Pachú *et al*. (2023); Tian *et al*. (2023); Li *et al*. (2024); Zhang *et al*. (2024)
Microfluidic devices	Emerging technologies for culturing	Precise control over microenvironment. Real-time observation. Can be scaled for high-throughput.	Simplified model compared with soil. No information on associated roots.	Studying hyphal growth, branching, and foraging strategies under controlled conditions. Investigating responses to different environmental factors.	μm–mm	Zhang *et al*. (2022); Takahashi *et al*. (2023); Hammer *et al*. (2024); Richter *et al*. (2024)
Electrical impedance tomography (EIT)	Visualizing mycorrhizal fungi and associated root structures	Non-destructive. *In situ* measurements. Relatively inexpensive. Provides temporal data (changes over time).	Cannot directly detect mycorrhizal hyphae (too fine). Provides information on root systems, not individual hyphae.	Studying how mycorrhizal colonization influences 3D root architecture over time. High-throughput screening of plant responses to mycorrhiza.	cm	Corona-Lopez *et al*. (2019); Liu *et al*. (2022); Han *et al*. (2023); Ain *et al*. (2024); Michels *et al*. (2024)
Positron emission tomography (PET)	Tracking nutrient exchange and metabolic processes	Non-destructive. 3D visualization of biochemical processes *in vivo*. High sensitivity (nano/picomolar). Can track radiotracers over time.	Requires specialized radionuclides and scanners. Medical scanners may not be ideal for plants. 18F has a short half-life	Tracking nutrient (e.g. sugars, amino acids, potentially lipids) and water movement in plants and mycorrhizal fungi.	mm–cm	Cameron *et al*. (2006); Jacquemyn *et al*. (2021); Komarov and Tai (2022); Metzner *et al*. (2022); Miyoshi *et al*. (2023a, b); Geisler *et al*. (2023); Lekberg *et al*. (2024); Brooks *et al*. (2024); Tausta *et al*. (2024)
Portable PET/MRI scanners	Field application of mycorrhizal fungi imaging	*In situ* measurements. Non-destructive.	Under development. May have lower resolution than lab-based scanners that result in the study of plant over fungal tissues.	Assessing plant stress and potentially mycorrhizal interactions in the field.	mm–cm	Veresoglou and Rillig (2012); Antonecchia *et al*. (2024); Blystone *et al*. (2024); Zhang *et al*. (2024)
Portable root imagers	Field application of mycorrhizal fungi imaging	*In situ* measurements. Can monitor development over time.	Limited to root observation, may not directly visualize hyphae.	Characterizing ectomycorrhizal morphotypes and monitoring their development in the field.	mm–cm	Thiem *et al*. (2023)
Transparent soil media (SAP)	Emerging technologies for culturing	Allows visualization of mycorrhizal fungi, non-sterile, simple.	Difficult to scale. Less control than other culturing systems.	Imaging of hyphae in near-soil conditions.	mm–cm	Paré *et al*. (2022, 2024); Klein *et al*. (2024); Kokkoris *et al*. (2024)
Hyperspectral imaging	Field application of mycorrhizal fungi imaging	Can be used for large-scale assessment (e.g. satellite data). Can predict mycorrhizal association types and plant responses (e.g. phosphorus, drought).	Requires specialised equipment and data analysis. Coarse detail. Information on fungi is unavailable and inferred.	Mapping mycorrhizal distributions at landscape scales. Assessing plant responses to mycorrhizal inoculation.	m–km	Kong *et al*. (2020); Sousa *et al*. (2021; Sun *et al*. (2021)
